# A retrospective comparative study of clinical efficacy of percutaneous short segment pedicle screw fixation with or without screwing of the fractured vertebra with O-arm navigation

**DOI:** 10.1186/s12891-022-05069-3

**Published:** 2022-02-01

**Authors:** Xiaofeng Shao, Peng Peng, Peng Yang, Tian Xu, Zixiang Liu, Xi Hua, Xiaoyu Zhu, Zhonglai Qian, Huilin Yang, Haiqing Mao, Kangwu Chen

**Affiliations:** Department of Orthopedic Surgery, The First Affiliated Hospital of Soochow University, No. 188 Shizi Street, Suzhou, 215006 Jiangsu China

**Keywords:** O-arm, Thoracolumbar fractures, Injured vertebrae, Fracture fixation, Percutaneous pedicle screws

## Abstract

**Objective:**

To retrospectively analyze the short and long-term efficacies of O-arm-navigated percutaneous short segment pedicle screw fixation, with or without screwing of the fractured vertebra.

**Methods:**

A total of 42 patients who underwent O-arm-navigated percutaneous short segment pedicle screw fixation for the treatment of thoracolumbar fractures from February 2015 to December 2018 were selected for analysis. The patients were divided into two groups according to the surgical intervention they received: Group A received percutaneous short segment pedicle screw fixation with screwing of the fractured vertebra and Group B received percutaneous short segment pedicle screw fixation without screwing of the fractured vertebra. Radiographic analysis included Cobb angles and percentage of anterior vertebral height (AVH%). Clinical functional outcomes were assessed using the visual analog scale (VAS) for back pain and the oswestry disability index (ODI) scores.

**Results:**

No significant differences were observed in the operation time and intraoperative blood loss between the two groups (*P* > 0.05). The length of incision was statistically significantly different between the two groups (*P* < 0.05). There was no significant difference in Cobb angle and AVH% between the two groups before and after the surgery (*P* > 0.05). However, the Cobb angle and AVH% were both significantly larger in Group A than Group B at the final follow-up (*P* < 0.05). In terms of clinical outcomes, there were no statistically significant differences in VAS and ODI scores between the two groups (*P* > 0.05).

**Conclusion:**

In the short term, both minimally invasive treatments were safe and effective in treating thoracolumbar fracture. Although there was significant difference between the two groups in Cobb angle and vertebral body height at the last follow-up, the difference was small. Therefore, these specific parameters will be an important outcome measure in further investigations.

**Supplementary Information:**

The online version contains supplementary material available at 10.1186/s12891-022-05069-3.

## Introduction

With the rapid development of industry and extensive popularization of high-speed vehicles, the frequency of thoracolumbar fractures is greatly increased. People suffering from this type of fracture may incur large medical costs and long-time recovery, which vastly increases social burden. Thoracolumbar fractures are more common in men, with a male-to-female ratio of about 2:1 and a peak incidence between the age of 20 and 40 [1].

The thoracolumbar segment (T11-L2) is a transitional region between the relatively low-motion thoracic vertebra and relatively high-motion lumbar vertebra, where 50–70% of thoracolumbar fractures occurs [2]. Thoracolumbar fractures result in spinal instability and nerve damage and often require surgery to achieve sufficient decompression, vertebral height restoration and stability, while avoiding kyphosis, nerve damage, and have accelerated recovery. Posterior short segment fixation is a widely used surgical method for the treatment of thoracolumbar fractures. Traditionally, pedicle screws were only inserted above and below the injured vertebral body. Although this surgical procedure is shown to save the segmental motion of the vertebral body, poor surgical outcomes, such as, spinal nonunion, implant failure, and increased kyphosis are commonly reported [3, 4]. Since Dick et al. [5] proposed the concept of adding intermediate screws to the injured vertebrae, this has become a widely used method of correcting thoracolumbar fractures.

Compared to the traditional posterior open surgery, the minimally invasive percutaneous pedicle screw placement technique employs smaller incision, and has less bleeding, less dissection of paraspinal muscle tissue, less pain, and rapid postoperative recovery [6, 7]. In recent years, the emergence of navigation equipment like O-arm machine greatly reduced surgical trauma, shortened operation time, and markedly improved accuracy of surgery [8, 9].

This retrospective study analyzed the clinical and imaging information of patients who underwent O-arm assisted minimally invasive percutaneous pedicle screw insertion for thoracolumbar fractures at our hospital from February 2015 to December 2018 to establish the short-term efficacies of both surgical methods and compare their differences.

## Materials and methods

### Clinical data

**Fig. 1:**
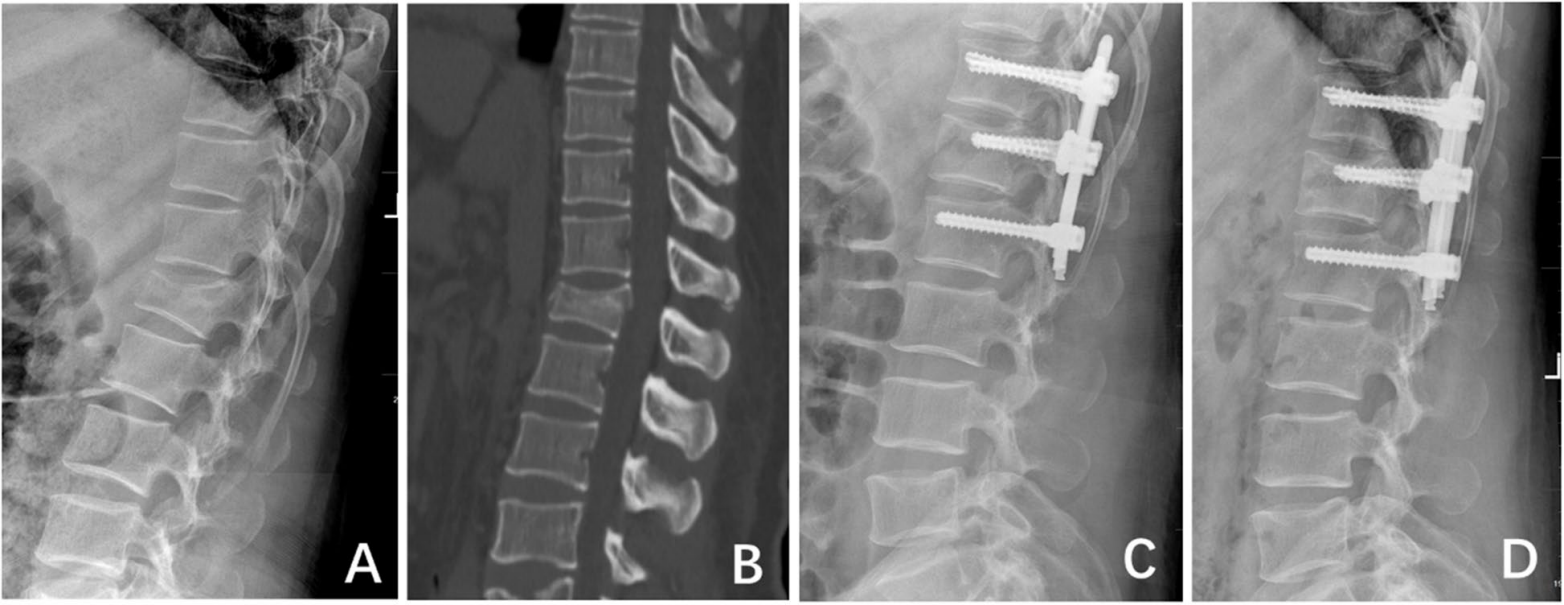
Lateral (**A**) radiograph and CT (**B**) of a 35-year-old male with L1 vertebral fracture (AO type B2). Receiving percutaneous short segment pedicle screw fixation without screwing of the fractured vertebra with O-arm navigation and the Cobb angle and AVH% get restoration one week (**C**) and one year (**D**) after the surgery

**Fig. 2:**
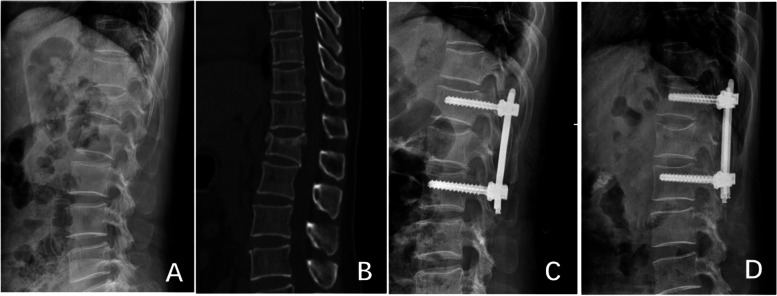
A 52-year-old female was diagnosed L1 vertebral fracture (AO type A1) by DR and CT (**A**, **B**). Percutaneous short segment pedicle screw fixation without screwing of the fractured vertebra with O-arm navigation was operated (**C**), and he got the satisfactory recovery (**D**)

The study was carried out with the approval of our institution’s ethics committee (IRB approval number is 2021–310). A consecutive series of 50 patients who underwent O-arm guided percutaneous minimally invasive pedicle screw fixation surgery to correct thoracolumbar fracture at our hospital from February 2015 to December 2018 were initially recruited for this study. After the inclusion and exclusion criteria were applied, 42 patients were included in our retrospective analysis. A detailed screening flowchart of our study is presented in Supplemental Fig. 1. Among the 42 participants, 21 accepted the O-arm guided percutaneous minimally invasive short segmental pedicle screw fixation with screwing of the fractured vertebra treatment (Group A) (Fig. 1, A1 ~ A4) and the rest accepted the O-arm guided percutaneous minimally invasive short segmental pedicle screw fixation without screwing of the fractured vertebra treatment (Group B) **(**Fig. 2**,** A1 ~ A4**)**. In our study, all patients who were hospitalized with thoracolumbar fractures had a detailed preoperative understanding of advantages and disadvantages of the two surgical methods. At the same time, patients were told that there was not enough evidence-based medicine to show which operation method was better. The operation method was decided by the spinal surgeons.

Our inclusion criteria were as follows: (1) a definite history of single-segment thoracolumbar fracture; (2) lack of nerve injury symptom and Grade E American Spinal Injury Association (ASIA) classification; (3) subjects received O-arm-navigated percutaneous short segment pedicle screw fixation with or without screwing of the fractured vertebra for treating injuries lasting less than 1 week; (4) follow-up duration was no less than 1 year. The exclusion criteria were as follows: (1) Patients had prior spinal surgeries or old fractures; (2) Patients had severe head trauma or internal organ injury or severe medical disease; (3) pedicle fracture, pathological fractures, or severe osteoporosis (bone mineral density < − 2.5); (4) patients with no valid follow-up information. Apart from the surgical methods, all participants received the same pre-, intra-, and postoperative treatments.

### Surgical techniques

#### Percutaneous short segment pedicle screw fixation with screwing of the fractured vertebra with O-arm navigation

Following successful general anesthesia, patient was placed in a prone position on the Jackson carbon fiber surgical bed, with the shoulders and buttocks well secured. A longitudinal 2.5 cm incision was made to expose the spinous process of the adjacent segment along the posterior median line, and the reference fixture was fixed to the spinous process. Next, the first scan of the O-arm was performed to obtain the intraoperative 3D images and transmit the image data to the Stealth Station navigation system, where high-resolution images in the axial, coronal, and sagittal were made on the screen. On the body surface, Passive Planar Probe was moved via 3D navigation system to determine skin incision where pedicle screws were to be inserted, then about 2 cm incision was made to make way for the placement of the registered Universal Drill Guide. The caudal and lateral angles of the Universal Drill Guide were adjusted to allow its projection on the sagittal and transverse navigation image to go through the pedicle axis. Subsequently, a channel was drilled using the Universal Drill Guide, and a total of six pedicle screws (WEGO, Shandong, China) were placed in the fractured vertebral body and its upper and lower vertebrae. The required connecting rod was measured and bent to connect the pedicle screws. Thus, the injured vertebra was propped up to restore the height of the fractured vertebra and the screw cap was tightened in the end. The pedicle screws and connecting rod were located accurately and fracture reduction was satisfactorily achieved, as evidenced by the O-arm scan. Lastly, the incision was closed.

#### Percutaneous short segment pedicle screw fixation without screwing of the fractured vertebra with O-arm navigation

This surgical method was similar to the upper surgical method, except for the following: during this surgery, no pedicle screws were inserted into the fractured vertebra, but the upper and lower vertebrae were still propped up to restore the height of the injured vertebra.

### After surgery

The patients were advised to actively move their legs the day after surgery. All patients began rehabilitation 2 days after surgery under the guidance of physician and were provided thoracolumbar rigid braces for 3 months. The patients were followed for at least 1 year.

### Clinical and radiological measurement

Imaging evaluation index included Cobb angle and percentage of anterior vertebral height (AVH%). The measurement of injured vertebral Cobb angle was as follows: a vertical line was made extending the upper vertebra superior endplate till it connected with the lower vertebra subjacent endplate extension on the lateral thoracic lumbar spine DR, the angle between the two perpendicular lines was the Cobb angle of the injured vertebra. Method of AVH% measurement was the anterior vertebral height of the injured vertebra / the mean value of the anterior vertebral height of the upper and lower adjacent segments × 100%. All fractures were classified according to the AO classification systems. The clinical evaluation indexes included intraoperative bleeding, operation time, and incision length. In addition, the visual analogue scale (VAS) and Oswestry disability index (ODI) scores were evaluated postoperatively and 1 year after the surgery.

### Statistical analysis

SPSS 20.0 statistical was used for statistical analysis, the comparison of the numerical data in these two groups adopted independent samples T test and paired sample T test was used for comparison in the same group. Pearson’s Chi-square test and the Fisher exact test were used for categorical data. *P* < 0.05 showed statistically significant difference.

## Results

### Demographic data

All participants were divided into two groups, based on the surgical method. Twenty-one patients who underwent percutaneous short segment pedicle screw fixation with screwing of the fractured vertebra were classified as Group A, and 21 patients who underwent percutaneous short segment pedicle screw fixation without screwing of the fractured vertebra served as Group B. Group A consisted of 10 males and 12 females, and the mean age was 53.33 ± 6.80 years, whereas Group B consisted of 12 males and 9 females, and the mean age was 54.05 ± 11.32 years. No significant statistical differences were detected in gender, age, trauma causes, fracture site, and type of fracture between the two groups (*P* > 0.05). Patient demographics of both groups are presented in Table 1.

**Table 1:** Patient demographic and injury details

Variable	Group A (*n* = 21)	Group B(*n* = 21)	*P* value
Gender (male/female)	10/11	12/9	0.537
Age (years)	53.33 ± 6.80	54.05 ± 11.32	0.805
Segment [n (%)]			0.745
T11	3 (14.3%)	3 (14.3%)	
T12	3 (14.3%)	3 (14.3%)	
L1	10 (47.6%)	12 (57.1%)	
L2	5 (23.8%)	3 (14.3%)	
Fracture type [n (%)]			1.000
A1	11 (52.4%)	12 (57.1%)	
A2	1 (4.8%)	0 (0.0%)	
A3	8 (38.1%)	7 (33.3%)	
B2	1 (4.8%)	2 (9.5%)	
Trauma cause [n (%)]			0.626
Falling injury	10 (47.6%)	9 (42.9%)	
Traffic injury	10 (47.6%)	10 (47.6%)	
Heavy bruise injury	1 (4.8%)	2 (9.5%)	

### General results

In group A, the surgical time was 160.24 ± 52.84 min, amount of bleeding was 99.76 ± 32.15 mL, and incision length was 11.95 ± 2.06 cm. In group B, the surgery time was 153.955 ± 28.15 min, amount of bleeding was 100.38 ± 23.00 mL, and incision length was 10.38 ± 1.36 cm. In terms of clinical indicators, there was no significant statistical differences in the operation time and intraoperative bleeding between the two groups (*P* > 0.05), but the surgical incision in Group A was longer than Group B (11.95 ± 2.06 vs. 10.38 ± 1.36 *P* = 0.006, Table 2). All patients underwent surgery successfully. There were no intraoperative complications, such as, abdominal aortic injury or cerebrospinal fluid leakage in either group. During the follow-up period, there were no neurological impairment symptoms or implant-related complication in patients from either group.

**Table 2:** Operation values

Variable	Group A (*n* = 21)	Group B (*n* = 21)	*P* value
Operation time (min)	160.24 ± 52.84	153.955 ± 28.15	0.633
Incision length (cm)	11.95 ± 2.06	10.38 ± 1.36	**0.006**
blood loss (ml)	99.76 ± 32.15	100.38 ± 23.00	0.619

Bold values indicate significant differences between two groups, *p* < 0.05

### Clinical outcomes

In Group A, the postoperative VAS score was 2.6 ± 0.5, and was recorded at 1.6 ± 0.5 at the last follow-up. In Group B, the postoperative VAS score was 2.4 ± 0.5 after surgery, and was recorded at 1.6 ± 0.5 at the last follow-up. There were no significant differences in VAS scores at different stages between the groups (*p* > 0.05, Table 3).

**Table 3:** Comparison of radiological and clinical data between groups

Variable	Group A (*n* = 21)	Group B (*n* = 21)	*P* value
Cobb angle (°)			
Pre-operative	12.81 ± 4.25	14.33 ± 6.37	0.368
Post-operative	4.76 ± 2.86^a^	6.62 ± 5.00^a^	0.147
Last follow-up	6.52 ± 3.04^a^	9.48 ± 5.38^a^	**0.035**
AVH% (%)			
Pre-operative	59.13 ± 11.88	64.51 ± 15.76	0.219
Post-operative	94.64 ± 10.22^a^	93.12 ± 15.97^a^	0.716
Last follow-up	87.51 ± 10.67^a,b^	78.99 ± 15.13^a,b^	**0.041**
VAS score			
Post-operative	2.6 ± 0.5	2.4 ± 0.5	0.367
Last follow-up	1.6 ± 0.5	1.6 ± 0.5	0.760
ODI (%)			
Post-operative	46.7 ± 15.2	48.1 ± 14.9	0.775
Last follow-up	5.5 ± 1.4	5.5 ± 1.5	0.915

Bold values indicate significant differences between two groups, *p* < 0.05

*AVH%* percentage of anterior vertebral height

^a^ Compared to pre-operation, *P* < 0.05

^b^ Compared with post-operation, *P* < 0.05

In Group A, the postoperative ODI score was 46.7 ± 15.2 after surgery, and was recorded at 5.5 ± 1.4 at the last follow-up. In Group B, the postoperative ODI score was 48.1 ± 14.9 after surgery, and was recorded at 5.5 ± 1.5 at the last follow-up. There were no significant differences in ODI at different stages between the groups (*p* > 0.05, Table 3).

### Radiographic outcomes

In Group A, the Cobb angle improved from 12.81 ± 4.25 prior to surgery to 4.76 ± 2.86 (significant, *p* < 0.001) post surgery, and was recorded as 6.52 ± 3.04 at the last follow-up. The AVH% increased from 59.13 ± 11.88 prior to surgery to 94.64 ± 10.22 (significant, p < 0.001) post surgery, and was recorded as 87.51 ± 10.67 at the last follow-up. In Group A, the differences in Cobb angle between postoperative and final follow-up values were not statistically significant (*P* > 0.05), whereas the differences in AVH% between postoperative and final follow-up values showed statistically significant (94.64 ± 10.22 vs. 87.51 ± 10.67, *P* < 0.001).

In Group B, the Cobb angle improved from 14.33 ± 6.37 prior to surgery to 6.62 ± 5.00 (significant, *p* < 0.001) post surgery, and was recorded at 9.48 ± 5.38 at the last follow-up. The AVH% increased from 64.51 ± 15.76 prior to surgery to 93.12 ± 15.97(significant, p < 0.001) post surgery, and was recorded at 78.99 ± 15.13 at the last follow-up. In Group B, the differences in Cobb angle between postoperative and final follow-up values were not statistically significant (*P* > 0.05), whereas the differences in AVH% between postoperative and final follow-up values showed statistically significant (93.12 ± 15.97 vs. 78.99 ± 15.13, *P* < 0.001).

There were no statistically significant differences in the fractured vertebra Cobb angle between the two groups before and 1 week after the surgery (*P* > 0.05). However, the Cobb angle 1 year after the surgery was maintained better in Group A than Group B (6.52 ± 3.04 vs. 9.48 ± 5.38 *P* = 0.035). There was no difference in AVH% between the two groups either before operation or 1 week after operation (P > 0.05), while AVH% between the two groups was statistically significant 1 year after surgery (87.51 ± 10.67 vs. 78.99 ± 15.13, *P* = 0.041).

## Discussion

In general, surgical treatment is the first choice in correcting thoracolumbar fracture with neurological deficits. However, there is no unified standard for treating thoracolumbar burst fractures without neurological deficits. Some scholars agreed that thoracolumbar burst fractures without nerve injury can achieve satisfactory clinical results via strict conservative treatment [10, 11]. However, Denis et al. [12] reported that surgical treatment can markedly enhance restoration of vertebral height and correct spinal deformity for thoracolumbar burst fractures without neurological symptoms. Moreover, the recovery of long-term neurological function in patients also fares better with surgery than with conservative treatment.

Traditional short-segment pedicle screw fixation without intermediate screws for thoracolumbar fractures achieves satisfactory results, but develops complications, such as, loss of postoperative correction and high incidence of screw breakage over time [13]. Ye et al. [14] compared clinical and radiological outcomes of 44 patients after short-segment pedicle screw instrumentation with or without intermediate screws for treating thoracolumbar fractures. The results revealed that the injured vertebra fixation group had a better curative effect than traditional short-segment pedicle screw instrumentation. In addition to the real-time correction of the injured vertebra during surgery, the long-term corrective effect of using intermediate screws surgery is also very idea [15, 16]. Farrokhi et al. [17] showed that the pedicle screw fixation with screwing of fractured vertebra achieves satisfactory reduction of the injured vertebrae, and allows for good correction of the Cobb angle, AVH, and vertebral height loss. In a meta-analysis [18] examining clinical curative effects of 310 patients, it was found that combined intermediate screw fixation technique was associated with better maintenance of the Cobb angle and the height of the injured vertebrae at follow-up. Moreover, the internal fixation failure rate was lower, but the operation time and intraoperative bleeding of the intermediate screw fixation group was slightly higher than the without intermediate screw fixation group.

However, pedicle screws fixation in the aforementioned studies was mainly performed by traditional open surgery. New minimally invasive techniques, such as, percutaneous screw placement provide a novel idea for the treatment of thoracolumbar fracture. Therefore, the aim of this study is to compare the safety and effectiveness between percutaneous short-segment pedicle screw fixation with or without screwing of the fractured vertebra assisted with O-arm navigation.

Generally, in injured vertebral four pedicle screw fixation surgery, the injured vertebral space is indirectly opened via force between the upper and lower vertebral body using the traditional nail rod system. The intra- and postoperative clinical effect of this surgery may be satisfactory in the short term, but in the long term, it results in the loss of correction and internal fixation failure, due to the four pedicle screw fixation (being a double plane fixation) inducing simultaneous quadrilateral and suspension effects [19]. Dick et al. reported that the additional pedicle screw placement in the injured vertebra vastly improves the biomechanical stability of the screw rod system by reducing the stress of each pedicle screw and by supporting the anterior column [5]. Segmental fixation with additional screws at the level of the fracture can therefore alter the original double plane fixation into a three-plane fixation by introducing the injured vertebra pedicle screws and avoiding both quadrilateral and suspension effects. During this procedure, the injured vertebra is directly thrust forward, thus promoting kyphosis reduction of the injured vertebra. In addition, the increased screws enhance construct stiffness and disperse stress, which, in turn, prevents internal fixation failure, and shields the fractured vertebral body from anterior loads. Mahar et al. examined the biomechanical properties of L1–3 in six specimens of human corpse. They adopted the L2 segment as a simulative damage section, and tested the outcomes of fractured vertebra screwing versus non-fractured vertebra screwing [20]. Based on their results, the biomechanics of the fractured vertebra screwing group was significantly stronger than the non-fractured vertebra screwing group. Bolesta et al. simulated the L2 vertebral body burst fracture on calf spine and conducted a biomechanical test [21]. This test revealed that the stability of the fixed segment can be increased by an average of 68% in the fractured vertebra screwing group, which is similar to the long-segment fixation procedure. Saglam et al. who conducted a study on four groups of patients receiving 4-segment cross-injury vertebra fixation, 3-segment cross-injury vertebra fixation, 4-segment cross-injury vertebra fixation, and 5-segment cross-injury vertebra fixation, also observed similar results [22].

In this study, no significant differences between the groups regarding VAS pain and ODI were seen at follow-up. We confirmed that both groups had relatively similar clinical effects in terms of restoration and pain relief, which meant that adding an intermediate screw into the fractured vertebral body did not result in increased postoperative pain. The length of incision in Group A showed longer in comparison with that in the Group B. Although the comparison of the incision length showed significant difference, the difference was small. This is likely to be ascribable to two additional pedicle screws inserted into the fractured vertebrae in Group A, which requires more intraoperative exposure [23]. However, no significant differences were observed in the operation time between the two groups, which mean it had little effect on the operation time.

Postoperative incision pain and discomfort after internal fixation were alleviated 1 year after the surgery in both groups of patients. However, the Cobb angle and the AVH% of Group A was statistically different from Group B 1 year after surgery, indicating that the ability to retain Cobb angle and AVH% was somewhat less in Group B. This may be because the injured vertebra fixation in Group A patients directly exerted forward force on the injured vertebra and significantly increased stability of the short-segment pedicle screw fixation. In the long term, this resulted in enhanced distraction effect of the upper and lower vertebra as well as the nail rod system, which, in turn, augmented the stability of the internal fixation, relative to the non-injured vertebra fixation in Group B.

There are some limitations in this study. First, this study was a retrospective study, the operation options for the patients mainly based on treatment experience of spinal surgeons, which might have selection bias. However, no significant differences were found in the radiological and clinical data between the both groups. Second, due to the strict inclusion criteria and need for at least 1 year follow-up, the number of cases meeting the requirements was relatively small. In addition, only one-year postoperative follow-up was collected in this study, and the results of longer follow-ups were not included. Further investigations are therefore necessary to evaluate longer follow-ups and determine the long-term efficacy of these interventions in treating thoracolumbar fractures.

## Conclusion

In summary, the percutaneous short-segment pedicle screw fixation with or without screwing of the fractured vertebra are both safe and effective in the treatment of thoracolumbar fractures. Although we found a significant difference between the both groups in Cobb angle and vertebral body height at the final follow-up, the statistical difference was small. Further investigations are required to verify the results of our study.

## Supplementary Information

**Additional file 1 Figure 1**. Flow Diagram.

## Data Availability

The datasets used and/or analyzed during the current study available from the corresponding author on reasonable request.

## Abbreviations

VAS: Visual analogue scales
AVH: Anterior vertebral height
ODI: Oswestry disability index
