# Supplementary material for: A retrospective comparative study of clinical efficacy of percutaneous short segment pedicle screw fixation with or without screwing of the fractured vertebra with O-arm navigation
Source: BMC Musculoskelet Disord. 2022 Feb 1;23:108. doi: 10.1186/s12891-022-05069-3 (PMC8808771; doi:10.1186/s12891-022-05069-3)
Supplement: Supplementary file 1 — Additional file 1 Figure 1. Flow Diagram. [file 12891_2022_5069_MOESM1_ESM.docx]

**Flow Diagram**

Cases excluded in order (n=8)

1. Follow-up less than 1 year or no valid follow-up information (n=2)
2. Previous injury or surgery (n=1)
3. Pathological fracture (n=1)
4. Osteoporosis (BMD<-2.5) (n=1)
5. Other injuries requiring surgery(n=2)
6. Severe medical disease(n=1)

Patients those received O-arm-navigated percutaneous posterior short-segment fixation for single level thoracolumbar fracture between February 2015 to December 2018 (n=50)

Underwent fixation without screwing of the fractured vertebra: group B (n=21)

Underwent fixation with screwing of the fractured vertebra: group A (n=21)

Patients those met the inclusion and exclusion criteria (n=42)
